# Involvement of reactive oxygen species in lanthanum-induced inhibition of primary root growth

**DOI:** 10.1093/jxb/erw379

**Published:** 2016-10-07

**Authors:** Yang-Yang Liu, Ru-Ling Wang, Ping Zhang, Liang-liang Sun, Jin Xu

**Affiliations:** Key Laboratory of Tropical Plant Resources and Sustainable Use, Xishuangbanna Tropical Botanical Garden, Chinese Academy of Sciences, Menglun, Mengla, Yunnan 666303, China

**Keywords:** Arabidopsis, lanthanum, primary root growth, reactive oxygen species, root system architecture.

## Abstract

Although lanthanum (La) has been used as an agricultural plant growth stimulant for approximately 50 years, high concentrations are toxic to plants. Despite significant advances in recent years, the mechanisms underlying the effects of La on root system development remain unclear. Here, we report that a high concentration of La inhibits primary root (PR) elongation and induces lateral root (LR) development. La results in cell death in PR tips, thereby leading to the loss of meristematic cell division potential, stem cell niche activity, and auxin distribution in PR tips. Further analysis indicated that La induces reactive oxygen species (ROS) over-accumulation in PR tips. Reduction in ROS accumulation partially alleviated the inhibitory effects of La on PR elongation by improving cell survival in PR tips and thereby improving meristematic cell division potential and auxin distribution in PR tips. We also found ROS to be involved in La-induced endocytosis. Genetic analyses supported the described phenotype. Overall, our results indicate that La affects root growth, at least partially, by modulating ROS levels in roots to induce cell death in PR tips and subsequent auxin redistribution in roots, leading to remodeling of the root system architecture.

## Highlight

ROS is involved in lanthanum-modulated root development by inducing cell death in the root tips of primary roots.

## Introduction

Rare earth elements (REEs) are relatively plentiful in the Earth’s crust (He *et al.*, 2005). Chien (1917) first reported that REEs improve the physiological activities of *Spirogyra*. Since the 1970s, REEs, particularly lanthanum (La), have been widely used in agriculture as plant growth stimulants because they promote crop growth (Hu *et al.*, 2004; Feng *et al.*, 2013). Indeed, REEs protect plants against certain plant diseases and stress conditions (Wang *et al.*, 2014). A low concentration of La^3+^ relieves plant growth inhibition, improves leaf water potential and water content, and increases soluble protein and proline contents while decreasing malondialdehyde contents under osmotic or salt stress (Feng *et al.*, 1999; Xu *et al.*, 2007). La^3+^ also enhances the antioxidant potential of wheat seedlings in response to lead stress by increasing antioxidant enzyme activities (Pang *et al.*, 2002). He *et al.* (2005) found that La improved cadmium (Cd) tolerance in *Lactuca sativa* by up-regulating expression of a phytochelatin synthase gene, *LsPCS1*, as well as phytochelatin levels in both the leaf and root.

Studies on the physiological roles of REEs in plants have achieved a number of advances in recent years. Several mechanisms that explain the effects of REEs have been proposed, including REE-mediated changes in antioxidative enzyme activities, photosynthesis efficiency, mineral nutrient uptake capacity, and phytohormonal balance (Ruíz-Herrera et al., 2012; Wang *et al.*, 2014). Wang *et al.* (2014) showed that REEs enter plant cells via endocytosis. REEs can also activate endocytosis in plant cells, which may be the cellular basis underlying the action of REEs in plants. La^3+^ has been used as a Ca^2+^ channel blocker, which subsequently disrupts abscisic acid (ABA) signaling and inhibits cyclin-dependent kinases and phospholipase D (Polya *et al.*, 1987; Hagenbeek *et al.*, 2000; Gampala *et al.*, 2001; Ruíz-Herrera *et al.*, 2012). Therefore, it is of fundamental importance to elucidate the molecular mechanisms underlying REE-mediated modulation of signaling pathways in plant cells.

The widespread use of REEs has caused their over-accumulation in soil and water (Wang *et al.*, 2011; Biasioli *et al.*, 2012; Wang *et al.*, 2014). In addition, physiological studies have revealed that REEs have dual effects on plant growth, with low concentrations of REEs improving the growth and yield of crops and high concentrations of REEs having a harmful effect (Wang *et al.*, 2014). Nonetheless, the molecular mechanisms underlying plant growth and development in response to a high concentration of REE remain poorly understood.

The root system architecture (RSA) of a plant has a crucial function in the uptake of nutrients and water and contributes to plant adaptations to abiotic stresses, and thus is also important for plant growth. Root growth and development is a complex process modulated by a variety of phytohormones and signaling molecules, with auxin playing a central role (Wang *et al.*, 2009). The maintenance of a normal auxin concentration and a steep auxin gradient in root tips is essential for this hormone to regulate stem cell differentiation, gravitropic responses, and lateral organ initiation (Laskowski *et al.*, 2008; Shi *et al.*, 2015). REEs inhibit PR growth and improve root hair and LR development, thereby modulating RSA. Ruíz-Herrera *et al.* (2012) found that reduced expression of the auxin-responsive *DR5:GUS* reporter in tPR tips might be responsible for inhibiting PR growth in Arabidopsis treated with gadolinium (Gd), another REE. However, the mechanisms responsible for the observed inhibition in root growth at high REE concentrations remain unclear.

The growth of PR depends on synergetic activity of cell division, elongation, and differentiation in the PR meristem (Tian *et al.*, 2014). Root stem cell niche activity and meristematic cell division potential are two crucial determinants of root growth (Liu *et al.*, 2016). The quiescent center (QC) plays a role in maintaining the identities of the surrounding stem cells (Scheres, 2007; Dinneny and Benfey, 2008). Both the *PLETHORA* (*PLT*) pathway and the *SHORT ROOT* (*SHR*)*/SCARECROW* (*SCR*) pathway regulate stem cell niche activity and QC identity. *SHR* activates the expression of *SCR* together with *WOX5* to regulate the balance between the QC identity and the root stem cell division and differentiation, and auxin plays a key role in the process (Aida *et al.*, 2004; Tian *et al.*, 2014; Liu *et al.*, 2016).

Reactive oxygen species (ROS) are important signaling molecules involved in modulating plant growth and development in response to environmental cues (Foreman *et al.*, 2003; Iglesias *et al.*, 2010; Bashandy *et al.*, 2010; Cheng *et al.*, 2011). However, high levels of ROS induce oxidative damage and subsequent cell death, and therefore repress plant growth and development. Although La^3+^ induces ROS accumulation and affects cellular redox signaling in plants (Feng *et al.*, 1999; Xu *et al.*, 2007), whether and how La^3+^-induced ROS accumulation affects RSA has yet to be explored. In the present study, we investigated the role of ROS in La-induced RSA remodeling. Our results show that ROS plays a role in La-mediated PR growth inhibition. The potential mechanisms involved in this process are discussed.

## Materials and methods

### Plant growth and chemical treatments

The Arabidopsis ecotype Columbia was used in this study. The following transgenic lines were used: *DR5:GUS*, *DR5:GFP*, *ABD2:GFP*, *PLT1:PLT1-GFP*, *SHR:SHR-GFP*, *QC25:GUS*, *CYCB1;1:GUS*, *WOX5:GUS*. The mutant lines used in this study include *rbohD* and *rbohF*. The seeds were surface sterilized for 5 min with 5% bleach, washed five times with sterile water, incubated for 2 days at 4 °C in the dark and plated onto agar medium containing half-strength Murashige and Skoog (MS) medium (Sigma-Aldrich) at pH 5.75 and supplemented with 1% agar and 10% sucrose. The seedlings were grown in a growth chamber maintained at 22 °C under a 16/8 h light/dark cycle. The plates were placed in a vertical position. Five-day-old seedlings were transferred to plates supplemented with various chemical treatments (lanthanum nitrate (Sangon), catalase (CAT), and KI) and grown for an additional 2–4 days.

### GUS staining

The GUS staining solution contained 50 mM sodium phosphate buffer at pH 7.0, 0.5 mM potassium ferricyanide, 0.5 mM potassium ferrocyanide, 10 mM EDTA, and 1 mg ml^–1^ 5-bromo-chloro-3-indolyl-β-D-glucuronide. Seedlings harboring a GUS reporter gene were incubated at 37 °C in staining solution for 3–5 h. The fluorescence intensity was quantified using ImageJ software.

### Measurement of ROS production

Endogenous ROS levels in root meristems were visualized using the specific ROS fluorescent probe 2,7-dichlorofluorescein diacetate (DCFH-DA) (Beyotime). Seedlings were incubated at 37 °C in 10 μM staining solution for 5 min, washed twice and then viewed under a Leica laser scanning confocal microscope (excitation, 488 nm; emission, 530 nm). The fluorescence intensity was quantified using ImageJ software.

For localizing H_2_O_2_ produced by Arabidopsis roots, treated roots were immersed in 1 mg ml^–1^ 3-diaminobenzidine (DAB)-HCl (pH 3.8) for 5 h and cleared by boiling in alcohol (95%, v/v) for 5 min. Photos were taken using a Carl Zeiss imaging system.

### Fluorescence microscopy

Green fluorescent protein (GFP) lines were observed with a confocal laser scanning microscope (Zeiss) according to the manufacturer’s instructions. The excitation and emission wavelengths were 488 and 520 nm.

### Phenotypic analysis

Seedlings were grown in a vertical position. After transfer to plates supplemented with various components, root growth was recorded every day at the same time. After 4 d of treatment, root lengths were measured and statistically analysed. Initiation of lateral root primordia (LRPs) was quantified in roots using the *DR5:GUS* reporter line. The four LRP developmental stages were classified as follows: up to three cell layers (stage A); more than three cell layers but not emerged (stage B); emerged lateral roots (LRs) <0.5 mm in length (stage C); and emerged LRs >0.5 mm in length (stage D) (Zhang *et al.*, 1999). Only mature LRs (>0.5 mm) were recorded as LRs.

### Quantitative reverse-transcription polymerase chain reaction analysis

We used whole seedlings for analysis of auxin biosynthesis-related gene expression and roots for analysis of the LR-related and ROS-related gene expression. Tissues were collected for total RNA isolation using TRIzol reagent (TaKaRa) according to the manufacturer’s instructions. Reverse transcription was then performed using PrimeScript^TM^ RT Reagent Kit with gDNA Eraser (TaKaRa). A quantitative PCR assay was performed using a LightCycler 480II (Roche) apparatus with UItraSYBR Mixture (CWBIO). The PCR assay was performed in 96-well plates as follows: incubation at 95 °C for 10 min for complete denaturation and 45 cycles at 95 °C for 10 s, 60 °C for 20 s, and 72 °C for 20 s. *ACTIN2* (AT3G18780) and *EF1a* (AT5G60390) were used as internal controls for quantitative reverse-transcription polymerase chain reaction (qRT-PCR) normalization with GeNorm (Czechowski *et al.*, 2005). The qRT-PCR analysis of each gene was performed on three biological replicates, including duplicates for each. The relative transcript levels for each sample were determined and averaged over the six replicates. The specific primers for each gene are listed in supplemental Supplementary Table S1 at *JXB* online.

### Quantification of IAA

Five-day-old Arabidopsis seedlings were exposed to 150 μM La(NO_3_)_3_ for 2 d, and the root IAA content was quantified according to Gao *et al.* (2014) and Liu *et al.* (2015). Approximately 0.1 g (fresh weight) of roots was collected and immediately frozen in liquid nitrogen. After extraction, endogenous IAA was purified, methylated in a stream of diazomethane gas and resuspended in 100 μl of ethyl acetate. The endogenous IAA content was analysed by gas chromatography–mass spectrometry (GC/MS).

### Nutrient content analysis

Five-day-old seedlings grown in 1/2 MS were treated with 150 μM La for 2 d. The treated seedlings were immersed for 2 h in a solution containing 1 mM EDTA and then thoroughly rinsed eight times with distilled water. The samples were oven dried at 75 °C for 72 h. The dried plant seedlings were ground and digested in concentrated nitric acid for 2–3 d at room temperature. The samples were then boiled for 1–2 h until completely digested. After adding 4 ml of Millipore-filtered deionized water and briefly centrifuging the solution, the contents of Mn, Zn, Cu, Fe, K and Ca were determined using inductively coupled plasma atomic emission spectroscopy (ICP-AES). Each experiment was repeated six times.

### Statistical analysis

At least 20 roots were analysed for each treatment, and all experiments were repeated at least three times. The results are presented as the mean±SEM. Tukey’s test (*P*<0.01) was used for statistical analyses.

## Results

### La inhibits PR growth by inducing cell death in PR tips

**Fig. 1.**
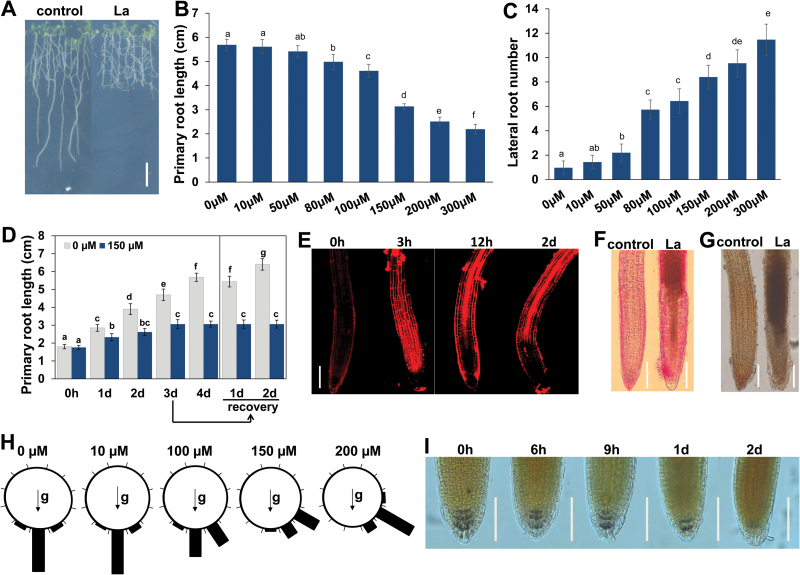
Effects of La on root system development in Arabidopsis. (A–C) Five-day-old Col-0 seedlings grown on 1/2 MS medium were treated with 0–300 μM La(NO_3_)_3_. (A) Image of seedlings grown on 150 μM La(NO_3_)_3_-supplemented medium for 4 d. Bar, 1 cm. (B, C) The primary root growth (B) and the number of lateral roots (C) were measured after 4 d of treatment. (D) Five-day-old Col-0 seedlings grown on 1/2 MS medium were treated with 150 μM La(NO_3_)_3_ for 1–4 d; after 3 d of treatment, the seedlings were re-transferred to normal 1/2 MS medium for 1–2 d. The error bars represent the SEM; *n*=60. Different letters indicate significantly different values (*P*<0.05 by Tukey’s test). (E) Image of PI staining of 5-day-old seedlings exposed to 150 μM La(NO_3_)_3_ for 3 h, 12 h, and 2 d. Bar, 50 μm. (F, G) Image of ruthenium red (F) and potassium permanganate (G) staining of 5-day-old seedlings exposed to 150 μM La(NO_3_)_3_ for 12 h. Bar, 100 μm. (H) Gravity responses of wild-type Col-0 roots at 2 d after reorientation of 90° to horizontal in the presence of 150 μM La(NO_3_)_3_. *n*=60. Each gravity-stimulated root was assigned to one of twelve 30° sectors. The length of each bar represents the percentage of seedlings showing the direction of root growth within that sector. (I) I_2_–KI staining images of seedlings treated without or with 150 μM La(NO_3_)_3_ for periods of up to 2 d. Bar, 50 μm. (This figure is available in color at *JXB* online.)

To examine the effects of La on root system growth, we transferred 5-day-old Arabidopsis seedlings germinated on 1/2 MS plates to new plates supplemented with different concentrations of La. The seedlings continued to grow for 4 d, and primary root (PR) growth and lateral root (LR) number were measured. With increasing La concentrations, PR growth was inhibited, and the LR number markedly increased from 10 to 300 μM (Fig. 1A–C). To further explore the effect of La on LR formation, we also analysed the initiation of LRP. As shown in Supplementary Fig. S1A, LRP initiation was increased in all four stages following exposure to 10–300 μM La. Consistent with the increased number of LRs, expression of three key marker genes involved in LR development, *LBD16*, *LBD29*, and *IAA14* (Lavenus *et al.*, 2013), also showed elevated expression in La-treated roots (Supplementary Fig. S1B). These results indicated that the La-induced inhibition of PR growth and increase in the number of LR occurred in a dose-dependent manner. Because treatment with 150 μM La induced an approximately 50% decrease in PR growth and did not result in plant death, we selected this concentration for further study.

To analyse whether La-repressed PR growth is a temporary growth-inhibitory effect, 5-day-old Arabidopsis seedlings were transferred to fresh plates supplemented with 150 μM La. PR growth was markedly inhibited by the treatment, with complete growth cessation after 3 d (Fig. 1D). After 3 d of treatment with 150 μM La, the seedlings were re-transferred to normal 1/2 MS plates, though PR growth did not recover when the stress was removed (Fig. 1D).

Therefore, we further tested the possibility that La-induced PR growth cessation might be caused by root death. To this end, we performed propidium iodide (PI) staining to examine cell death in roots. We found that 3 h of treatment with La caused rapid cell death in root tips, especially in the transition zone and elongation zone (Fig. 1E). We next examined whether exposure to La also affects cell differentiation in these zones. Ruthenium red staining and potassium permanganate (KMnO_4_) staining revealed that La induced extensive mucification of the cell wall and lignification in the elongation zones (Fig. 1F, G), indicating that La induced premature root tip differentiation.

One of the typical phenotypes caused by La treatment is the loss of gravitropism (Fig. 1H). As amyloplasts in the columella cells of roots have been proposed to play a critical role in sensing gravity in roots (Sun *et al.*, 2008), we used I_2_–KI staining to test the hypothesis that La treatment alters amyloplasts in the columella cells of roots. Our results showed that with prolonged treatment, La induced a substantial reduction in the number of amyloplasts in root columella cells, with amyloplasts almost completely absent in columella cells after 2 d of treatment (Fig. 1I). These results suggested that the reduction in the number of columella cell amyloplasts was responsible for the loss of gravitropism in response to treatment with La.

**Fig. 2.**
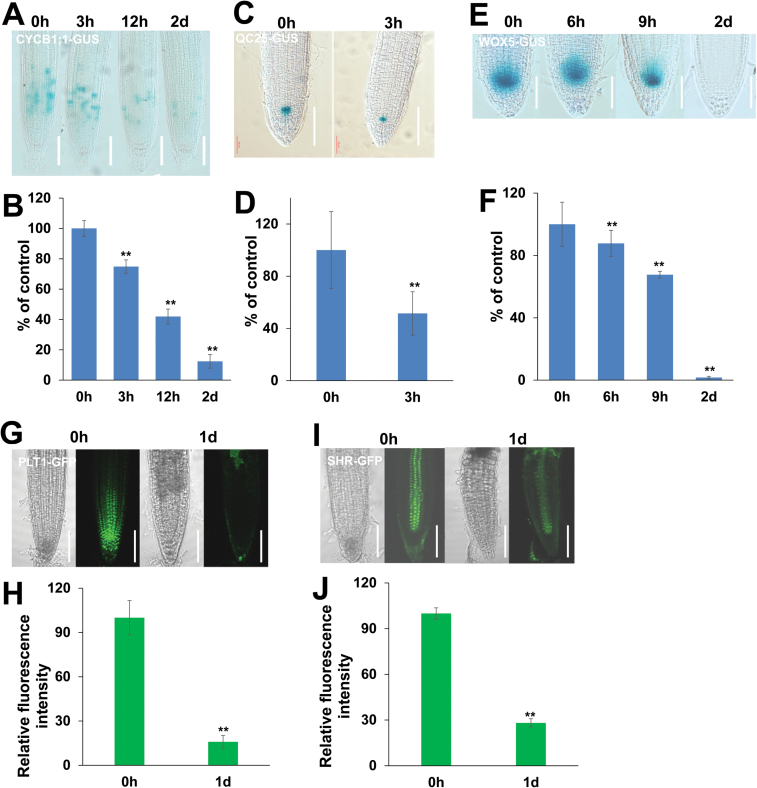
Effect of La on root meristem development. (A, B) Image of GUS staining (A) and the relative GUS activity (B) of 5-day-old *CYCB1;1:GUS* seedlings exposed to 150 μM La(NO_3_)_3_ for 3 h, 12 h, and 2 d. (C, D) Image of GUS staining (C) and the relative GUS activity (D) of 5-day-old *QC25:GUS* seedlings exposed to 150 μM La(NO_3_)_3_ for 3 h. (E, F) Image of GUS staining (E) and the relative GUS activity (F) of 5-day-old *WOX5:GUS* seedlings exposed to 150 μM La(NO_3_)_3_ for 6 h, 9 h, and 2 d. The GUS activity in the untreated roots was set to 100. Bar, 50 μm. (G, H) GFP fluorescence (G) and quantification of the *PLT1:GFP* fluorescence intensities (H) in the roots of 5-day-old *PLT1:GFP* seedlings exposed to 150 μM La(NO_3_)_3_ for 1 d. (I, J) GFP fluorescence (I) and quantification of the *SHR:GFP* fluorescence intensities (J) in the roots of 5-day-old *SHR:GFP* seedlings exposed to 150 μM La(NO_3_)_3_ for 1 d. The fluorescence intensity of the untreated roots was set to 100. Bar, 50 μm. *n*=60. The error bars represent the SEM. **P<0.01. (This figure is available in color at *JXB* online.)

To further confirm that La induces cell death in roots, we also investigated meristematic cell division potential and stem cell niche activity in root tips. We first analysed the meristematic cell division potential using a transgenic line expressing *CYCB1;1:GUS*, a marker used to monitor cell cycle progression (Colon-Carmona *et al.*, 1999). We found that GUS activity gradually decreased in the roots and was almost completely lost after 2 d of La treatment (Fig. 2A, B), indicating that La treatment impeded the cell cycle. We then examined stem cell activity using *QC25:GUS*, which is specifically expressed in the quiescent center (QC) (Sabatini *et al.*, 1999), and found GUS staining in the QC to be significantly reduced (Fig. 2C, D). Expression of WOX5 in the QC is critical for maintaining the stem cell niche (Sarkar *et al.*, 2007); therefore, we also assessed the pattern and progression of La-mediated WOX5 expression. Consistent with the results of *QC25:GUS* staining, the GUS activity of *WOX5:GUS* gradually decreased and almost completely disappeared after 2 d of La treatment (Fig. 2E, F). Because *PLETHORA* (*PLT*) acts in concert with *SHORT ROOT* (*SHR*) to control QC identity (Sabatini *et al.*, 1999), we examined the influence of La on PLT1 and SHR expression using *PLT1pro::PLT1-GFP* and *SHRpro::SHR-GFP* transgenic lines (Tian *et al.*, 2014) and found that expression of both reporters was markedly repressed in La-treated roots (Fig. 2G–J).

**Fig. 3.**
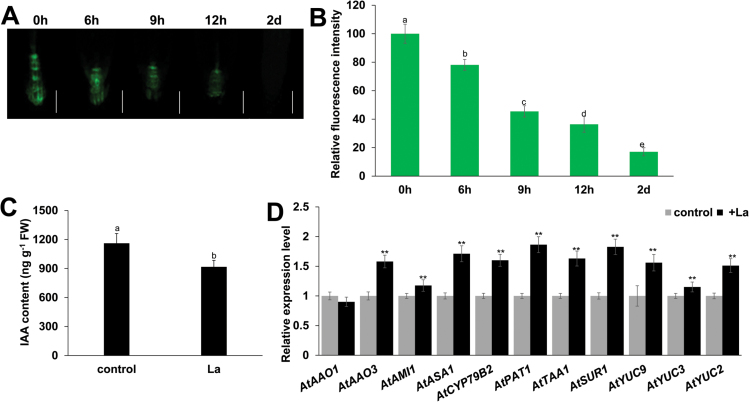
La represses auxin accumulation in PR tips. (A, B) GFP fluorescence (A) and quantification of the *DR5:GFP* fluorescence intensities (B) in the roots of 5-day-old *DR5:GFP* seedlings exposed to 150 μM La(NO_3_)_3_ for 6 h, 9 h, 12 h, and 2 d. The fluorescence intensity of the untreated roots was set to 100. Bar, 50 μm. (C) IAA contents in the roots of the wild-type seedlings treated with or without 150 μM La(NO_3_)_3_ for 2 d. (D) qRT-PCR analysis of the expression of auxin biosynthesis-related genes in wild-type Col-0 seedlings treated with or without 150 μM La(NO_3_)_3_ for 2 d. The expression levels of the indicated genes in the untreated roots were set to 1. The error bars represent the SEM. Different letters indicate significantly different values (*P*<0.01 by Tukey’s test). **P<0.01. (This figure is available in color at *JXB* online.)

Auxin plays a central role in modulating root system development. We thus investigated the effect of La-induced cell death on auxin distribution in root tips using the *DR5:GFP* and *DR5:GUS* marker lines. Five-day-old seedlings grown on 1/2 MS medium were transferred to fresh 1/2 MS medium with or without 150 μM La for periods of up to 2 d, and GFP fluorescence and GUS activity were then measured. Consistent with the observed cell death in root tips, prolonged La treatment significantly reduced expression of the two auxin reporters in root tips, and even completely repressed DR5 activity after 2 d (Fig. 3A, B; Supplementary Fig. S2A, B). Taken together, these data indicate that La induced cell death in roots, thereby leading to loss of meristematic cell division potential, stem cell niche activity, and auxin accumulation in PR tips, and subsequent cessation of PR growth.

Interestingly, we found that although La treatment repressed auxin distribution in PR tips, the GUS activity of *DR5:GUS* in LR tips was unaffected (Supplementary Fig. S2B). Therefore, we sought to determine whether La affects IAA levels in the root system. GC/MS analysis revealed a decrease in root IAA content by 17.5% after 2 d of treatment compared with untreated control (Fig. 3C). We next examined the transcript levels of genes encoding key enzymes in the auxin biosynthesis pathway using qRT-PCR. Interestingly, we found that La treatment increased the transcript levels of many IAA biosynthesis genes, including *ASA1*, *PAT1*, *SUR1*, *CYP79B2*, *TAA1*, *AMI1*, *YUC2*, *YUC3*, *YUC9*, and *AAO3* (Fig. 3D). This result is discussed below.

The root system is the primary organ by which plants uptake water and nutrients, and La alters RSA by inhibiting PR growth and increasing LR development. Thus, we examined nutrient contents in seedlings. ICP-AES analysis indicated that La treatment reduced calcium (Ca) levels but markedly increased the levels of iron (Fe), copper (Cu) and zinc (Zn). In contrast, the levels of potassium (K) and manganese (Mn) were unaffected (Table 1). These data indicated that La affected uptake and accumulation of elements in plants.

**Table 1.** Metal contents in Arabidopsis seedlings Five-day-old wild-type seedlings grown on 1/2 MS medium were treated with 150 μM La(NO_3_)_3_ for 4 d. The metal contents were determined as described in material and methods. *n*=6. The error bars represent the SEM. Different letters indicate significantly different values (*P*<0.01 by Tukey’s test).

	K (g kg^–1^)	Ca (g kg^–1^)	Fe (g kg^–1^)	Mn (g kg^–1^)	Cu (mg kg^–1^)	Zn (mg kg^–1^)
ck	31.41 ± 6.75 a	4.3 ± 0.03 a	0.57 ± 0.01 a	0.13 ± 0.001 a	3.2 ± 0.13 a	122.33 ± 0.44 a
La	30.56 ± 7.95 a	4.19 ± 0.03 b	0.68 ± 0.02 b	0.13 ± 0.001 a	4.37 ± 0.09 b	136 ± 2 b

### La induces ROS accumulation in root tips

**Fig. 4.**
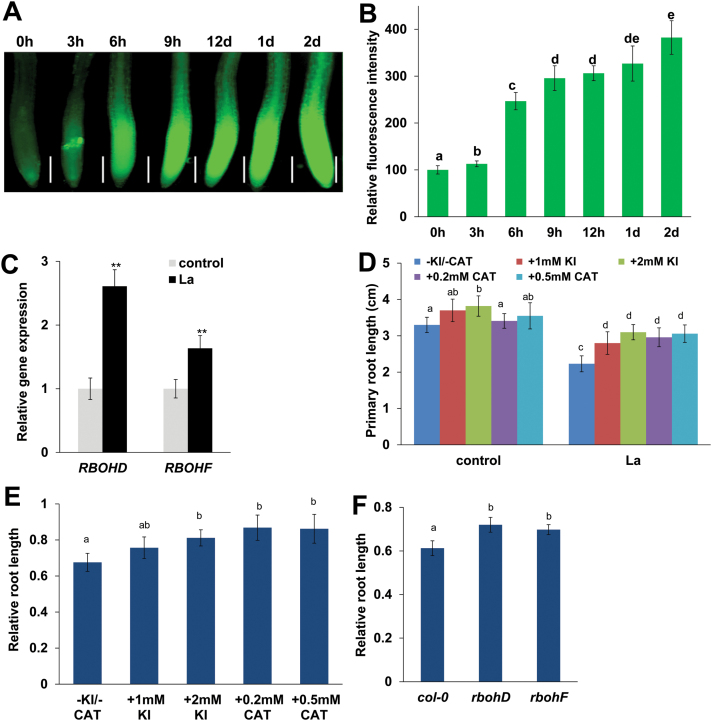
Involvement of ROS in the La-mediated inhibition of PR growth. (A, B) Detection of ROS production in the roots of 5-day-old wild-type seedlings exposed to 150 μM La(NO_3_)_3_ for periods of up to 2 d using the ROS-specific fluorescent probe DCFH-DA (A) and quantification of the ROS fluorescence intensities (B) in roots treated as in (A). Bar, 200 μm. The fluorescence intensity of the untreated roots was set to 100. (C) Quantitative RT-PCR analysis of *RBOHD* and *RBOHF* expression in the roots of the Col-0 seedlings treated with or without 150 μM La(NO_3_)_3_ for 2 d. The expression levels of the indicated genes in the untreated roots were set to 1. (D, E) Primary root length of Col-0 seedlings treated with or without 150 μM La(NO_3_)_3_ in the presence or absence of KI (1 or 2 mM) or CAT (0.2 or 0.5 mM) for 4 d (D). The data are presented relative to the La-untreated control values obtained from Col-0 seedlings in the presence or absence of KI (1 or 2 mM) or CAT (0.2 or 0.5 mM) for 4 d (E). (F) The relative root lengths of Col-0, *rbohD*, and *rbohF* seedlings treated with 150 μM La(NO_3_)_3_ are presented relative to the La-untreated control values obtained from Col-0, *rbohD*, and *rbohF* seedlings for 4 d. *n*=60. The error bars represent the SEM. Different letters indicate significantly different values (*P*<0.01 by Tukey’s test). **P<0.01. (This figure is available in color at *JXB* online.)

To investigate whether ROS signaling is involved in La-mediated PR growth inhibition, we measured ROS levels in PRs of La-treated seedlings using the ROS fluorescent probe DCFH-DA and DAB staining. As shown in Fig. 4A, B and Supplementary Fig. S3, La treatment induced ROS accumulation in PR tips. To further confirm that La induced this ROS accumulation, we performed qRT-PCR to estimate the transcript levels of two genes, *RBOHD* and *RBOHF*, that encode respiratory burst oxidase homologs for ROS production in La-treated roots (Kwak *et al.*, 2003). Consistent with the finding of increased ROS accumulation in roots, the qRT-PCR results revealed that La treatment significantly increased the levels of *RBOHD* and *RBOHF* transcripts in Arabidopsis roots (Fig. 4C).

### ROS is involved in La-mediated inhibition of PR growth

We next explored the physiological mechanisms underlying the effects of ROS on La-mediated PR growth by applying the ROS scavenger potassium iodide (KI) as well as catalase (CAT). CAT catalyses the dismutation of H_2_O_2_ to H_2_O and O_2_. KI, a scavenger of hydroxyl radicals (Chen and Schopfer, 1999), is also reported to scavenge H_2_O_2_, and its application to transgenic line 3*5S:UPB1-3YFP* can partially rescue H_2_O_2_ inhibition of root growth (Dunand *et al.*, 2007; Tsukagoshi *et al.*, 2010; Yuan *et al.*, 2013). La-induced accumulation of ROS was markedly inhibited in the presence of KI or CAT (Supplementary Fig. S4), and supplementation with KI or CAT alleviated the La-induced inhibition of PR growth (Fig. 4D, E). We next analysed PR growth in the ROS-deficient mutants *rbohD* and *rbohF* after La treatment. La-induced accumulation of ROS was markedly inhibited in *rbohD* and *rbohF* mutants (Supplementary Fig. S5), with a smaller degree of PR growth reduction than in La-treated wild-type plants (Fig. 4F).

**Fig. 5.**
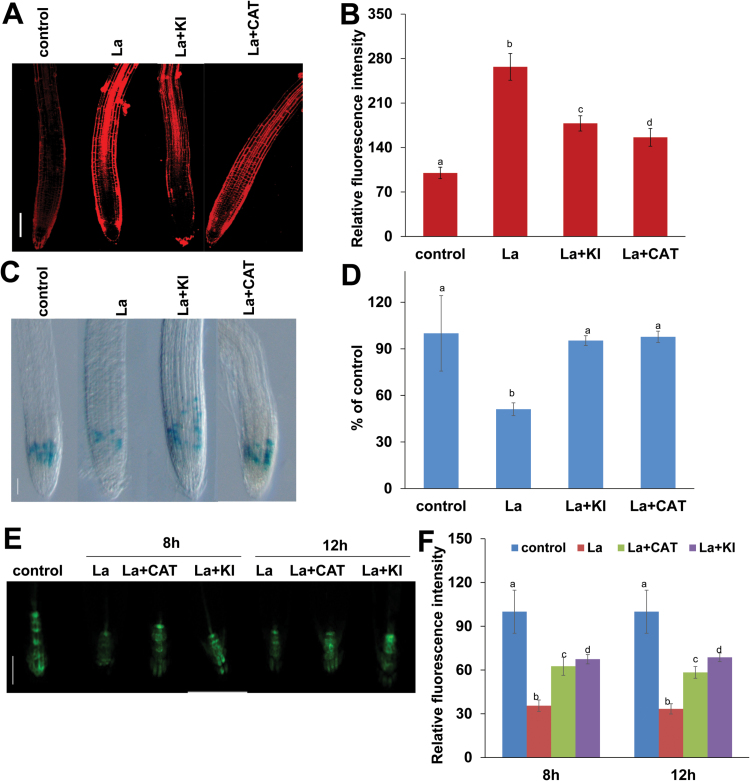
Reduction of ROS accumulation alleviated La-induced cell death in PR tips. (A, B) Images of PI staining in the Arabidopsis root cells from 5-day-old Col-0 seedlings treated with or without 150 μM La(NO_3_)_3_ in the presence or absence of 1 mM KI or 0.2 mM CAT for 12 h (A) and quantification of the PI fluorescence intensities (B) in roots treated as in (A). Bar, 100 μm. (C, D) Image of GUS staining (C) and the relative GUS activity (D) of 5-day-old *CYCB1;1::GUS* seedlings exposed to 150 μM La(NO_3_)_3_ in the presence or absence of 1 mM KI or 0.2 mM CAT for 12 h. Bar, 100 μm. (E, F) GFP fluorescence (A) and quantification of the *DR5:GFP* fluorescence intensities (B) in the roots of 5-day-old *DR5:GFP* seedlings exposed to 150 μM La(NO_3_)_3_ in the presence or absence of 1 mM KI or 0.2 mM CAT for 8 h and 12 h. Bar, 50 μm. The fluorescence intensity or GUS activity in the untreated roots was set to 100. *n*=60. The error bars represent the SEM. Different letters indicate significantly different values (*P*<0.01 by Tukey’s test). (This figure is available in color at *JXB* online.)

We also examined the effects of ROS on La-induced cell death in the roots using PI staining and demonstrated that the decrease in ROS production by KI or CAT reduced La-induced cell death in the roots (Fig. 5A, B). In addition, supplementation with KI or CAT resulted in additional GUS activity in the roots of the La-treated *CYCB1;1:GUS* marker line (Fig. 5C, D).

The above results indicated that decreased ROS accumulation reduced La-induced cell death in the roots. Thus, we speculated whether it could increase auxin distribution in root tips. Indeed, supplementation with KI or CAT prevented the decrease in DR5:GFP expression in PR tips (Fig. 5E, F). Taken together, these data indicated that reduced ROS production could alleviate cell death in roots and improve meristem cell division potential and auxin accumulation in root tips, thereby mitigating PR growth inhibition due to La.

### ROS is associated with La-induced endocytosis in Arabidopsis roots

**Fig. 6.**
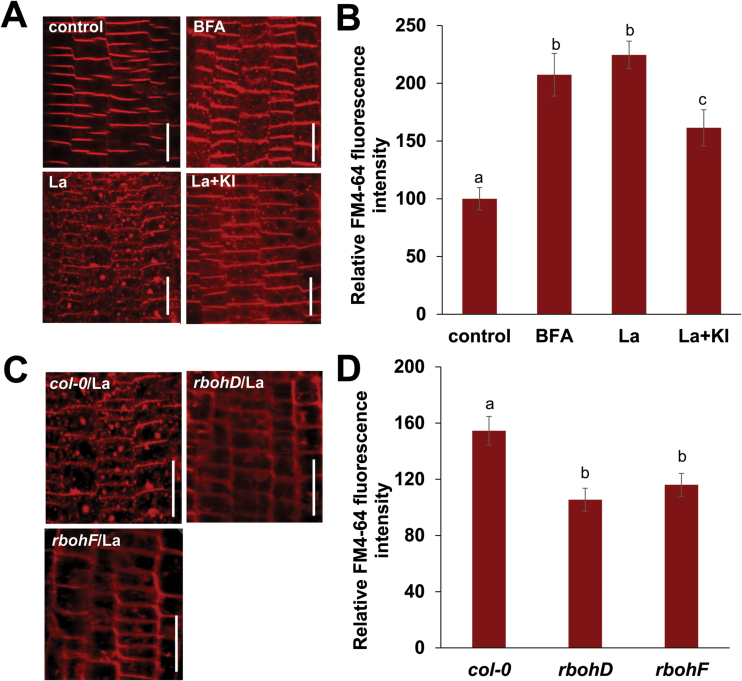
La-induced endocytosis in the root cells. (A, B) Images of FM4-64 staining in Arabidopsis root cells from 5-day-old Col-0 seedlings treated with or without 150 μM La(NO_3_)_3_, 50 μM BFA, or 150 μM La(NO_3_)_3_ plus 1 mM KI for 3 h (A) and quantification of the total FM4-64 fluorescence intensities (B). (C, D) Images of FM4-64 staining in Arabidopsis root cells from 5-day-old Col-0, *rbohD*, and *rbohF* seedlings treated with 150 μM La(NO_3_)_3_ for 3 h (C) and quantification of the total FM4-64 fluorescence intensities (D). Bar, 20 μm. The error bars represent the SEM. Different letters indicate significantly different values (*P*<0.01 by Tukey’s test). (This figure is available in color at *JXB* online.)

It has been observed that La activates endocytosis in plant cells, and this process might play a role in La-mediated plant growth and development (Wang *et al.*, 2014). We therefore investigated the effect of La on endocytosis in Arabidopsis roots. Upon treatment with brefeldin A (BFA), large aggregates characteristic of BFA bodies were formed, and they contained FM4-64. Exposure to La led to a significant increase in the endocytic uptake of FM4-64 dye in the root epidermal cells (Fig. 6A, B). A previous study revealed that actin microfilaments play a role in modulating endocytosis (Nagawa *et al.*, 2012). Therefore, we examined potential changes in the actin microfilaments in the La-treated roots using seedlings that express an *actin-binding domain 2* (*ABD2*)*:GFP* marker. We observed that treatment with La resulted in the reorganization and reorientation of the actin microfilaments in the cells of the transition zone in the root tips (Supplementary Fig. S6), indicating that La-induced endocytosis could be modulated by actin microfilaments.

To investigate the possible role of ROS in La-induced endocytosis, we examined the effect of KI on endocytosis in Arabidopsis root. Supplementation with KI partially alleviated La-induced endocytic uptake of FM4-64 dye in the root epidermal cells (Fig. 6A, B). In addition, genetic analyses indicated that La-induced endocytic uptake of FM4-64 dye in the root epidermal cells could be alleviated to a greater extent in the *rbohD* and *rbohF* mutants than in wild-type Col-0 plants (Fig. 6C, D). These results suggest that ROS are also involved in La-induced endocytosis.

## Discussion

Previous studies have shown that REEs, which serve as growth stimulants, improve the physiology of plants, but that high concentrations inhibit plant growth. These studies focused on the physiological effects of REEs on plant growth; however, the molecular mechanisms underlying REE growth modulation, particularly in the root system, have not been investigated to date. In this study, we found that 150 μM La resulted in significant PR growth repression as well as LR formation; however, plant death was not observed on the 1/2 MS medium until 3 weeks after treatment with La. These data are consistent with the results of previous studies that suggested that the modulation of RSA by REEs is unlikely to be related to plant toxicity because the formation of LRs and root hairs was normal in the presence of high concentrations of REEs (Kobayashi *et al.*, 2007; Ruíz-Herrera *et al.*, 2012). However, we also found that high concentrations of La result in remarkable cell death in PR tips. The meristematic cell division potential and stem cell niche activity, two main factors that affected root growth (Baluska *et al.*, 2010; Liu *et al.*, 2015), were markedly repressed and almost completely lost after 2 d of La treatment. Both the *SHR*/*SCR* pathway and the *PLT* pathway are involved in *WOX5*-mediated QC identity (Aida *et al.*, 2004; Blilou *et al.*, 2005; Grieneisen *et al.*, 2007; Galinha *et al.*, 2007; Dinneny and Benfey, 2008; Ding and Friml, 2010; Zhao *et al.*, 2014; Ji *et al.*, 2015). SHR and SCR are putative GRAS transcription factors that function in maintaining root stem cell niche activity and QC identity (Ji *et al.*, 2015). Parallel to the SHR/SCR pathway, the *PLT*s are essential for root stem cell niche patterning (Galinha *et al.*, 2007; Dinneny and Benfey, 2008; Zhao *et al.*, 2014). Consistent with the La-induced loss of the expression of WOX5:GUS and QC25:GUS, the expression of both the PLT1 and SHR reporters was also markedly repressed in the La-treated roots. These data further confirmed that La-induced cell death in PR tips resulted in loss of the meristematic cell division potential and stem cell niche activity and thereby cessation of PR growth.

Our results indicated that La treatment specifically induced cell death in PR tips, and thereby disrupted auxin transport and distribution, finally resulting in loss of auxin accumulation in PR tips. This lack of auxin accumulation in root tips also resulted in loss of gravitropism. Several lines of evidence support these conclusions. First, although La almost completely inhibited auxin distribution in PR tips, as indicated by DR5:GFP and DR5:GUS expression, GC/MS analysis indicated that La treatment reduced the IAA content in roots by only 17.5% compared with the untreated control. Second, qRT-PCR analysis showed that La treatment increased the expression of auxin biosynthesis-related genes in seedlings. Third, La treatment did not inhibit DR5 expression in LR tips, whereas it markedly promoted LR development. Taken together, our results indicated that La promotes root system development by terminating PR growth and mediating auxin redistribution in roots, thus promoting LR development. Future studies will explore whether La can regulate root system development by directly modulating auxin transport and responses.

RSA is important for nutrient and water uptake (López-Bucio *et al.*, 2003). Thus, we hypothesized that La-modulated RSA remodeling would affect nutrient levels, and our results indicated that La treatment increased the contents of Fe, Cu, and Zn in roots. These results showed that La-modulated RSA remodeling improved the uptake and accumulation of micronutrients and was beneficial for plant growth and development. However, further investigation is required to determine whether La can affect the uptake and accumulation of micronutrients by directly regulating the expression of metal transporters.

Consistent with previous reports that La^3+^ can be used as a Ca^2+^ channel blocker (Polya *et al.*, 1987; Hagenbeek *et al.*, 2000; Gampala *et al.*, 2001; Ruíz-Herrera *et al.*, 2012), we found that La treatment decreased Ca accumulation in Arabidopsis. Ca^2+^ is an important second messenger in both animals and plants. Therefore, it is reasonable to believe that La-induced changes in physiological and molecular phenotypes may occur through La-repressed Ca signaling and its interaction with other phytohormones and signaling molecules. However, further investigation is needed.

The involvement of ROS in plant responses to environmental cues and development processes has been widely reported. Indeed, ROS are important signal molecule that are involved in modulating plant growth and development; however, high levels of ROS-induced oxidative damage results in cell death. In this study, we found that La markedly induced over-accumulation of ROS in roots. Pharmacological and genetic approaches were also used in this study to provide evidence for the involvement of ROS in La-induced inhibition of PR growth. Treatment with the ROS scavenger KI and CAT significantly alleviated La-induced cell death in PR tips, improved meristematic cell division potential and auxin accumulation, and partially recovered La-induced PR growth inhibition. The PR growth in the *rbohD* and *rbohF* mutants was less sensitive to La than in the control condition. These data indicated that ROS over-accumulation is involved in La-induced cell death in root tips and subsequent PR growth inhibition. However, we found that reduction in ROS accumulation partially, but not completely, recovered La-induced PR growth inhibition, suggesting that pathways other than those involving ROS also participate in the process, which needs further elucidation.

La-induced endocytosis in plant cells has been reported, and the process might be responsible for La-mediated plant growth and development (Wang *et al.*, 2014). In the present study, we found that ROS are also involved in La-induced endocytosis. Nonetheless, additional studies are required to understand how endocytosis functions in La-mediated PR growth inhibition. Taken together, our results indicate that La inhibits PR growth by inducing cell death in PR tips, thereby altering auxin distribution in roots and causing subsequent RSA remodeling, and that ROS are involved in these processes. Such an understanding is helpful for crop cultivation and provides insights into the development of a root system necessary for plant adaptation by using the REEs.

## Supplementary data

Supplementary data are available at *JXB* online.

Table S1. List of the primers for qRT-PCR analysis of the genes.

Figure S1. La-induced LR development.

Figure S2. *DR5::GUS* staining.

Figure S3. DAB staining.

Figure S4. Detection of ROS production in the roots of seedlings exposed to La in the presence or absence of KI or CAT.

Figure S5. Detection of ROS in the roots of Col-0, *rbohD*, and *rbohF*.

Figure S6. GFP fluorescence in the roots of *ABD2::ABD2-GFP* seedlings.

Supplementary Data
