# Supplementary material for: Involvement of reactive oxygen species in lanthanum-induced inhibition of primary root growth
Source: J Exp Bot. 2016 Oct 7;67(21):6149–59. doi: 10.1093/jxb/erw379 (PMC5100026; doi:10.1093/jxb/erw379)
Supplement: Supplementary Data [file supp_67_21_6149__index.html]

Involvement of reactive oxygen species in lanthanum-induced inhibition of primary root growth — Involvement of reactive oxygen species in lanthanum-induced inhibition of primary root growth — Supplementary Data 

# Involvement of reactive oxygen species in lanthanum-induced inhibition of primary root growth

## Supplementary Data

Data files

- supplementary\_table\_S1\_figures\_S1\_S6.pdf - Supplementary Data
