# Supplementary material for: Involvement of reactive oxygen species in lanthanum-induced inhibition of primary root growth
Source: J Exp Bot. 2016 Oct 7;67(21):6149–59. doi: 10.1093/jxb/erw379 (PMC5100026; doi:10.1093/jxb/erw379)
Supplement: Supplementary Data [file supp_erw379_supplementary_table_S1_figures_S1_S6.pdf]

Supplemental files

Table S1 List of the primers for qRT-PCR analysis of the genes.

Gene	Primers
AtLBD29	5’gctaggcttcaagatcccatc3’<br>5’tgtgctgcttgttgctttaga3’
AtLBD16	5’tgaccctgtttatggatgtgtc3’<br>5’tgattgcaagaaagccacct3’
AtIAA14	5’GCAGAGGAGGCAATGAGTAGTG3’<br>5’GAGCATCCAGTCACCATCTTTG3’
AtCYP79B2	5’CACGATGATGCTCGCGAGACT3’<br>5’TCACTTCACCGTCGGGTAGAGA3’
AtASA1	5’ATGTCTTCCTCTATGAACGTAGC3’<br>5’ACAGCGGTAAATTGGTATAAGG3’
AtPAT1	5’ATGGTTATTGCGGTGGCGAC3’<br>5’ATCGTCGCCGACTCAATGTC3’
AtAMI1	5’CGGACTTACTCCAATGGCTCAG3’<br>5’GCTGCTGCAGGAGAACGCAACC3’
AtSUR1	5’GACCACCAAGGTGTTACAATCC3’<br>5’ATTATTGTGGCAGGGTCAGG3’
AtTAA1	5’CTCCAAGATCACAGGCCACGCTGGG3’<br>5’GACTCCTTAGACACACCAATCGAGTTC3’
AtYUC2	5’GGTGACACGGATCGGTTAGGGT3’<br>5’TGCCGAATAATGCATTACCCGT3’
AtYUC3	5’CTTGAGATTGATTCCGTTATTC3<br>5’GGAGAAGAAGTCGTTGTC3’
AtYUC9	5’ATCTTGCTAACCACAATG3’<br>5’CCACTTCATCATCATCAC3
AtAAO1	5’TGCCTGTTCCAGCAACAATG3’

	5’ TAAGCAGAACACCGCCATTG 3’
AtAAO3	5’GGAGTCAGCGAGGTGGAAGT3’<br>5’TGCTCCTTCGGTCTGTCCTAA3’
AtRBOHD	5’ATTACAAGCACCAAACCAG3’<br>5’TTCTCCGACCATCTCACTA3’
AtRBOHF	5’TCACAAATCAACGACGAGAGTT3’<br>5’CCCATCTTCATTCTTGTCCA3’

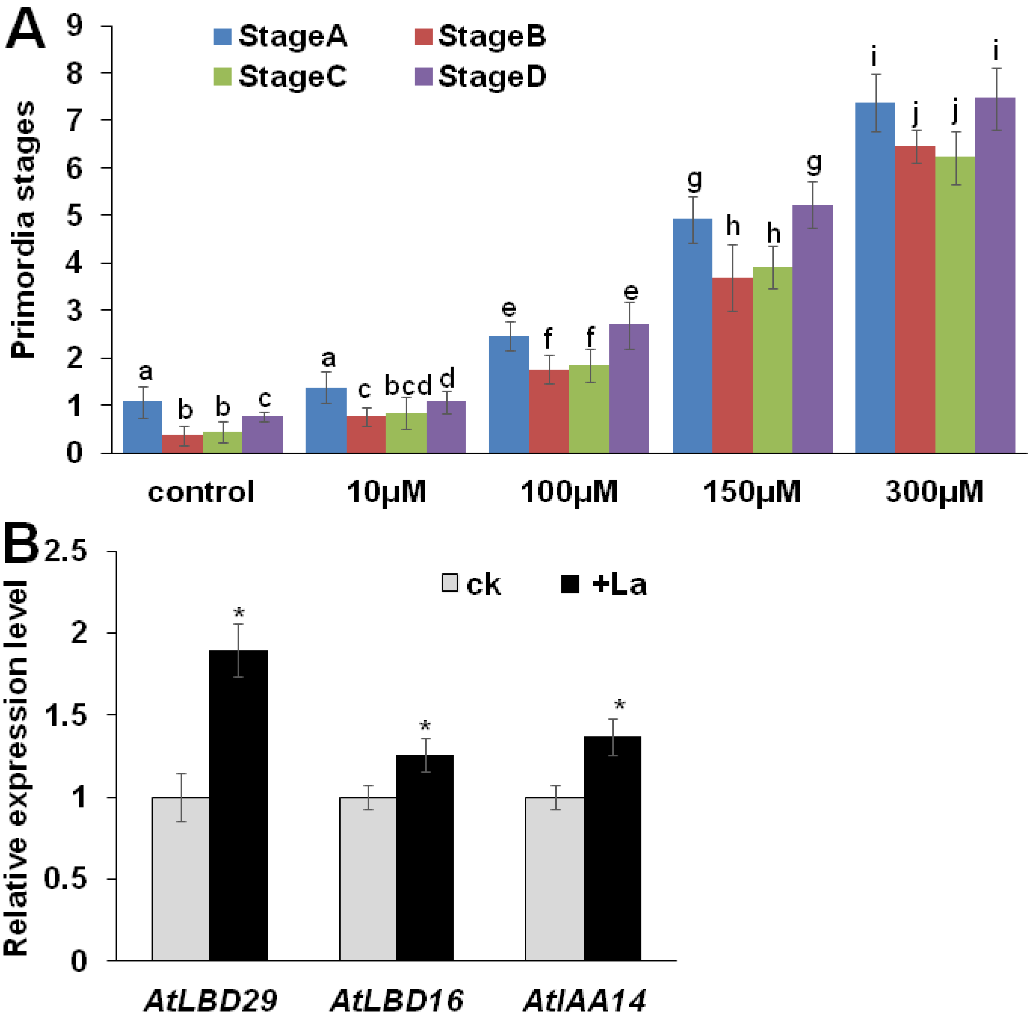

Fig. S1. Effect of La on LR development. (A) Five-day-old *DR5:GUS* seedlings grown in 1/2 MS medium were treated with 150 μM $La(NO_3)_3$ for 5 d, the lateral root primordia (LRP) initiation was measured. The four developmental stages of LRP were classified as follows: up to three cell layers (stage A); more than three cell layers but not emerged (stage B); emerged lateral roots (LRs) <0.5 mm in length (stage C); and emerged LRs >0.5 mm in length (stage D) (Zhang *et al.*,, 1999). Only mature LRs (>0.5 mm) were recorded as LRs. (B) Quantitative RT-PCR analysis of *AtLBD29*, *AtLBD16*, and *AtIAA14* expression in the roots of five-day-old *col-0* seedlings treated with or without 150 μM $La(NO_3)_3$ for 2 d. The expression levels of the indicated genes in the untreated roots were set to 1. The error bars represent the SEM. *p-value<0.01.

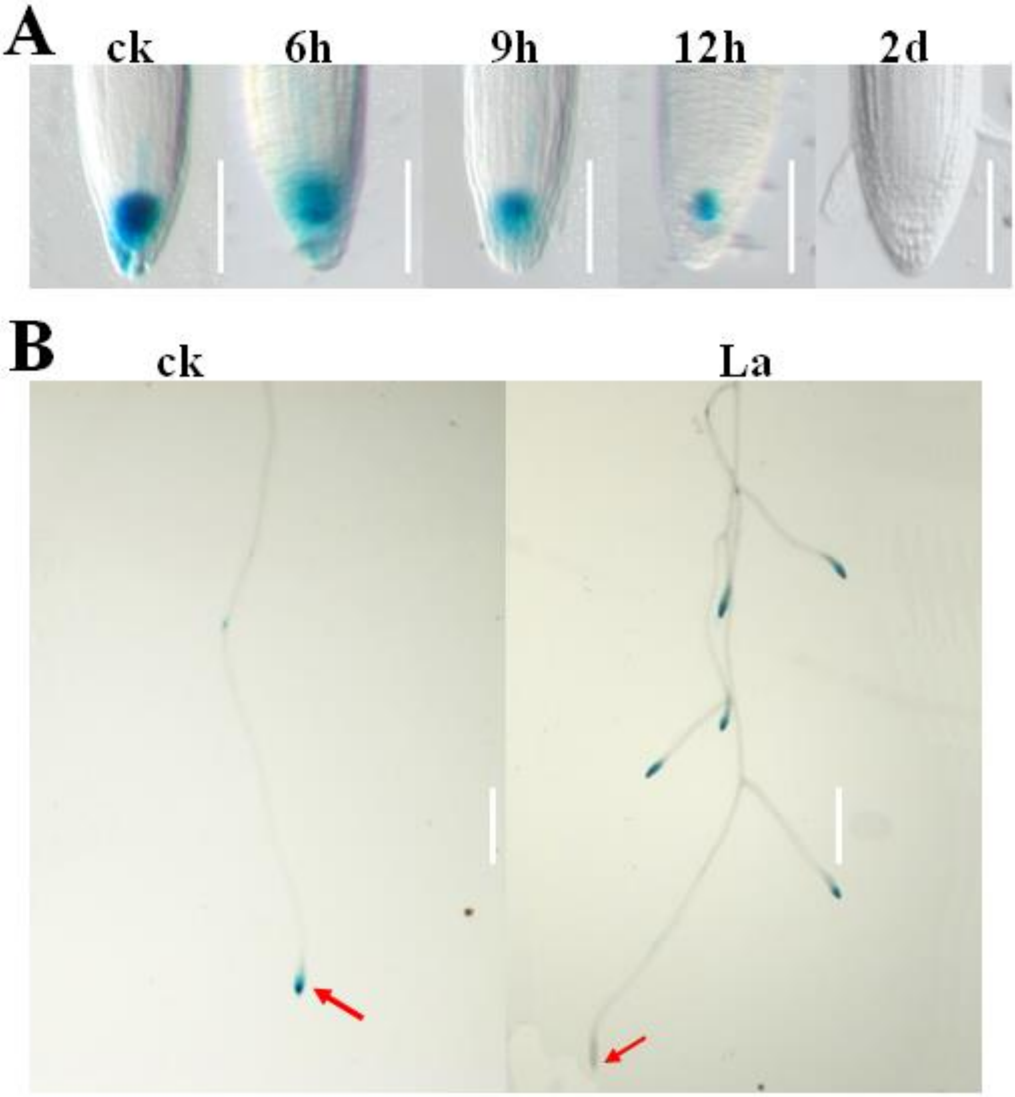

Fig. S2. (A) Image of GUS staining of the root tips of primary roots of 5-day-old *DR5::GUS* seedlings exposed to 150 μM $La(NO_3)_3$ for periods of up to 2 d. Bar, 100 μm. (B) Images of GUS staining of the lateral roots and primary roots. Bar, 1 mm.

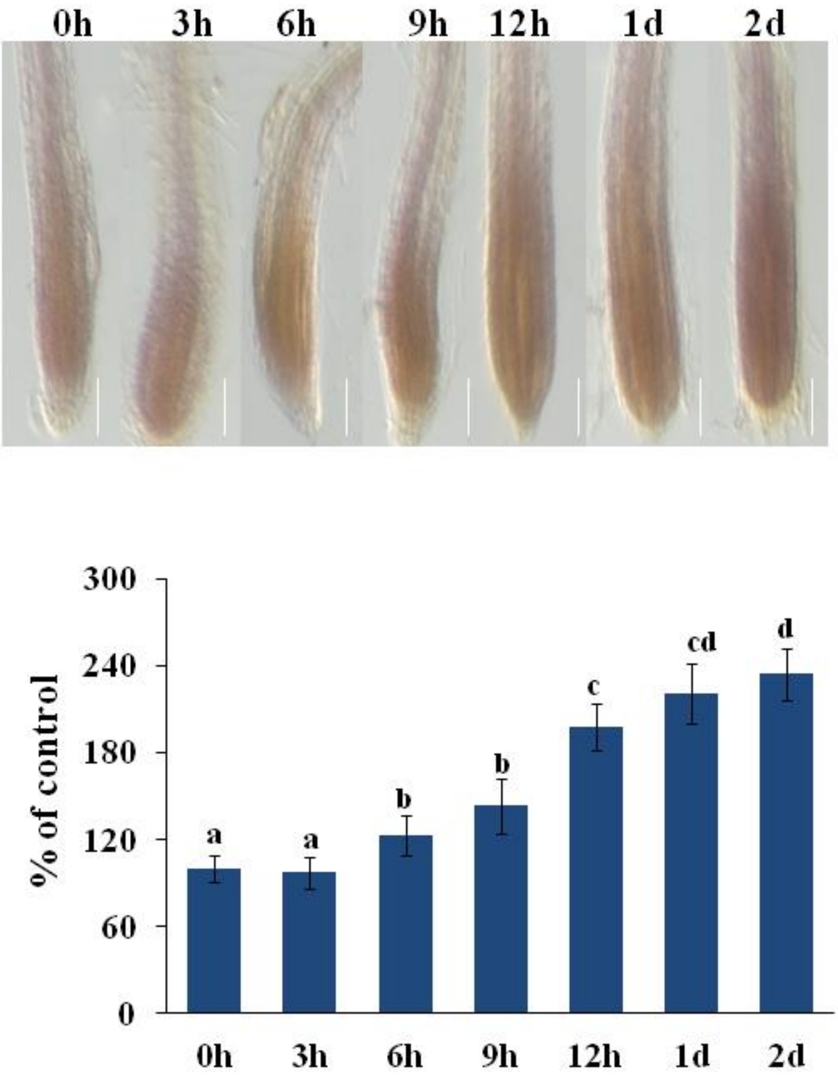

Fig. S3. Detection of ROS production in the roots of 5-day-old wild-type seedlings exposed to 150 μM $La(NO_3)_3$ for periods of up to 2 d using DAB staining. The error bars represent the SEM. Different letters indicate significantly different values ($P < 0.05$ by Tukey's test). Bar, 100 μm.

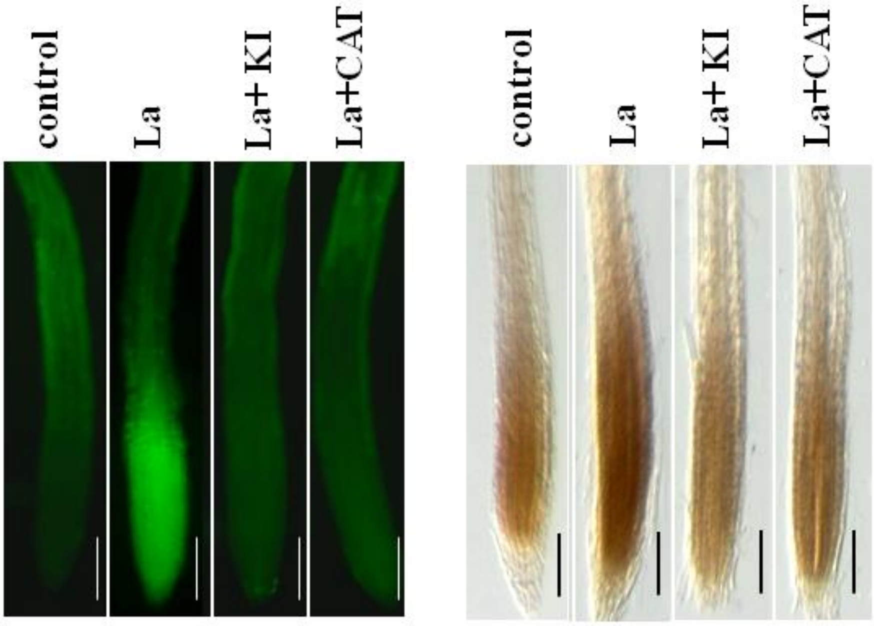

Fig. S4. Detection of ROS production in the roots of 5-day-old wild-type seedlings exposed to 150 μM $La(NO_3)_3$ for 6 h in the presence or absence of 1 mM KI or 0.2 mM CAT using the ROS-specific fluorescent probe DCFH-DA (left) and DAB staining (right). Bar, 100 μm.

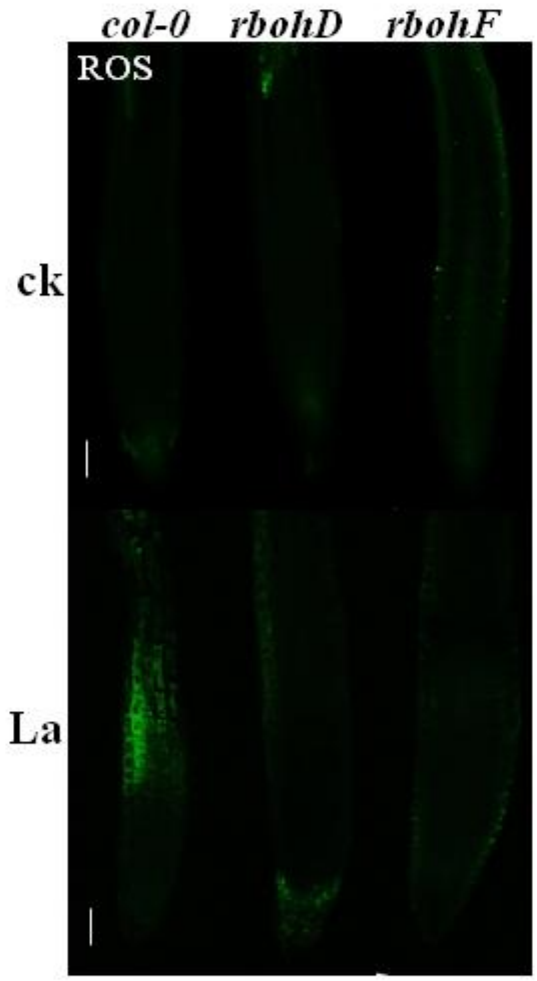

Fig. S5. Detection of ROS in the roots of 5-day-old *col-0*, *rbohD*, and *rbohF* seedlings treated with or without 150 μM $La(NO_3)_3$ for 12 h using the H_2O_2-specific fluorescent probe DCFH-DA. Bar, 50 μm.

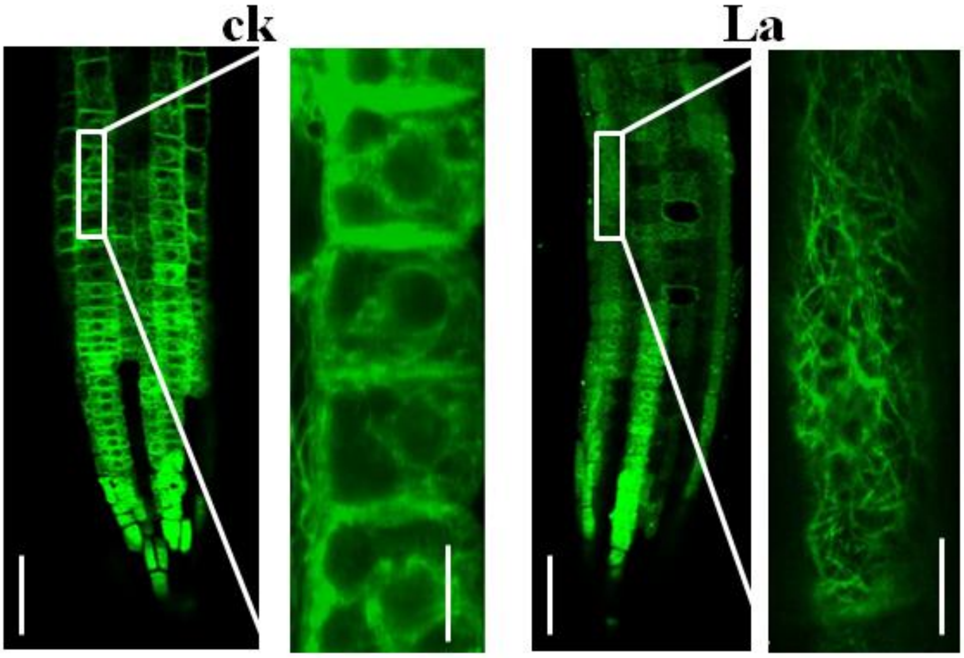

Fig. S6. GFP fluorescence in the roots of 5-day-old *ABD2::ABD2-GFP* seedlings exposed to 150 μM $La(NO_3)_3$ for 1 d.
